# Pleiotropic phenotypes of a *Yersinia enterocolitica flhD *mutant include reduced lethality in a chicken embryo model

**DOI:** 10.1186/1471-2180-8-12

**Published:** 2008-01-23

**Authors:** Megan K Townsend, Nathan J Carr, Jyoti G Iyer, Shelley M Horne, Penelope S Gibbs, Birgit M Prüß

**Affiliations:** 1 Department of Veterinary and Microbiological Sciences, North Dakota State University, Fargo, USA

## Abstract

**Background:**

The *Yersinia enterocolitica *flagellar master regulator FlhD/FlhC affects the expression levels of non-flagellar genes, including 21 genes that are involved in central metabolism. The sigma factor of the flagellar system, FliA, has a negative effect on the expression levels of seven plasmid-encoded virulence genes in addition to its positive effect on the expression levels of eight of the flagellar operons. This study investigates the phenotypes of *flhD *and *fliA *mutants that result from the complex gene regulation.

**Results:**

Phenotypes relating to central metabolism were investigated with Phenotype MicroArrays. Compared to the wild-type strain, isogenic *flhD *and *fliA *mutants exhibited increased growth on purines and reduced growth on N-acetyl-D-glucosamine and D-mannose, when used as a sole carbon source. Both mutants grew more poorly on pyrimidines and L-histidine as sole nitrogen source. Several intermediates of the tricarboxylic acid and the urea cycle, as well as several dipeptides, provided differential growth conditions for the two mutants. Gene expression was determined for selected genes and correlated with the observed phenotypes. Phenotypes relating to virulence were determined with the chicken embryo lethality assay. The assay that was previously established for *Escherichia coli *strains was modified for *Y. enterocolitica*. The *flhD *mutant caused reduced chicken embryo lethality when compared to wild-type bacteria. In contrast, the *fliA *mutant caused wild-type lethality. This indicates that the virulence phenotype of the *flhD *mutant might be due to genes that are regulated by FlhD/FlhC but not FliA, such as those that encode the flagellar type III secretion system.

**Conclusion:**

Phenotypes of *flhD *and *fliA *mutants are related to central metabolism and virulence and correlate with gene regulation.

## Background

The flagellar hierarchy in *Yersinia enterocolitica *is believed to be similar to *E. coli *with FlhD/FlhC constituting the master regulator [1] and FliA the sigma factor [2]. FlhD/FlhC is expressed from the *flhD *operon, that consists of the *flhD *and *flhC *genes [3]. The two proteins form a heterohexameric complex [4] and are referred to as class I in the three-tiered regulatory hierarchy of flagellar expression [for reviews, please, see [5-8]]. The FlhD/FlhC complex binds to upstream sequences of class II genes, including the *fliA *gene [9]. FliA is the sigma factor of the flagellar system [10] and is required for the expression of the class III flagellar genes [11].

A more detailed investigation of the flagellar hierarchy of *Y. enterocolitica *demonstrated FliA regulation of eight of the 15 flagellar operons [12]. The expression levels of the remaining seven operons were affected by FlhD/FlhC in a FliA independent manner. In accordance with the *E. coli *nomenclature, genes that were regulated by FliA were classified as class III [12]. It is noteworthy that the two operons that contain the chemotaxis genes, *motA *and *tar*, are both regulated by FliA, while the three operons that contain genes of the flagellar type III secretion system, *flhB, fliF *and *fliL*, are independent from FliA. Regulation of the three *Y. enterocolitica *flagellin genes, *fleA*, *fleB*, and *fleC*, by FliA had been previously shown [13,14].

In addition to their function as flagellar regulators, FlhD/FlhC and FliA are both global regulators that affect the expression of a large number of non-flagellar genes [for a review, please, see [15]]. FlhD/FlhC affects the expression levels of many metabolic genes [16], while FliA affects the expression levels of several plasmid-encoded virulence genes [12]. Genes with a large difference in expression between the wild-type cells and the *flhD *mutants were genes involved in carbamoylphosphate synthesis and degradation, a pathway that leads into the biosynthesis of pyrimidines and *hut *genes that are involved in the utilization of histidine [16]. It was concluded that FlhD/FlhC might be involved in the flux of nitrogen, using carbamoylphosphate as a checkpoint. Regulation of the plasmid-encoded virulence genes by FliA was most likely through inhibition of their transcriptional activator VirF [12].

In this study, we extend our phenotypic analysis of *Y. enterocolitica flhD *and *fliA *mutants. In agreement with our previous microarray analysis [12], wild-type cells grew better on pyrimidines as nitrogen sources. Interestingly, both mutants grew better on purines as carbon sources. In agreement with their higher expression of the *hut *genes, wild-type cells grew better on histidine.

Since FliA appeared to be a regulator of plasmid-encoded virulence genes [12], we needed an animal model to study the effect of flagellar proteins on virulence of *Y. enterocolitica*. A chicken embryo lethality assay (ELA) was previously developed to determine the pathogenic potential of *E. coli *strains [17]. This assay has been exhaustively studied [17-21]. The typical variability and proper statistical analysis for dichotomous data has been established using the logistic regression analysis and resulting odds ratios. This analysis compares the likelihood of death of any given embryo with a specific bacterial isolate. Although the ELA has been used primarily as an assay for comparing the pathogenic potential of avian *E. coli *isolates, its design was also intended to permit comparative virulence studies for isogenic mutants of the Enterobacteriacea [17]. For this work, the ELA was modified for *Y. enterocolitica *strains.

Dose response curves for the virulent 8081v isolate and the 8081c isolate that had been cured of the pYV virulence plasmid were obtained. Lethality of the 8081v isolate, as well as its isogenic *flhD *and *fliA *mutants were determined. Lethality caused by the *flhD *mutant was reduced relative to the 8081v strain, while the *fliA *mutation did not affect virulence in this model. The possibility of the reduced lethality of the *flhD *mutant being the consequence of a lacking flagellar type III secretion system is discussed.

## Results and discussion

### Mutants in *flhD *and *fliA *have pleiotropic phenotypes on carbon and nitrogen sources

Phenotypes of the 8081c *Y. enterocolitica *wild-type strain and its isogenic *flhD *and *fliA *mutants were investigated with Phenotype MicroArrays (PM; Biolog, Hayward, CA). PM1 plates contain carbon sources and PM3 plates contain nitrogen sources. To correlate the data with the previously described gene regulation [16], the experiments were performed at 25°C, 37°C, and 4°C. Growth patterns were compared between wild-type 8081c bacteria and their isogenic *flhD *or *fliA *mutant strains.

**Figure 1:**
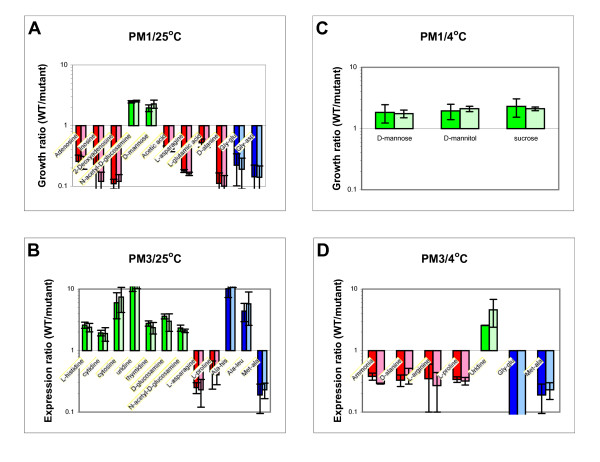
**Phenotype MicroArrays, nutrients**. Growth on sole carbon and nitrogen sources was determined with Phenotype MicroArrays (Biolog, Hayward, CA) for wild-type 8081c cells and their isogenic *flhD *and *fliA *mutants. Bacterial solutions in IF-0 were pipetted onto PM1 (carbon sources, Panels A and C) and PM3 (nitrogen sources, Panels B and D) plates. Plates were incubated at 25°C (Panels A and B) or 4°C (Panels C and D). Growth on all plates was determined as OD_630_. The background OD_630 _(obtained from well A1) was subtracted from the OD_630 _for each nutrient on each plate. Growth ratios are expressed as OD_630 _of the wild-type cells divided by OD_630 _of each of the mutant strains (darker bars, *flhD *mutant; lighter bars, *fliA *mutant) for each nutrient. Nutrients that provided better growth conditions for the wild-type cells are printed in green, nutrients that provided better growth conditions for the mutants are printed in red. Dipeptides are printed in blue. The experiment was performed three to four times and the mean of the population is presented. Error bars indicate one standard deviation (1 s).

Fig. 1 shows the determined phenotypes of the strains on carbon and nitrogen sources. At 25°C (Panel A), *flhD *mutants grew better on the purines adenosine, inosine, and 2-deoxyadenosine as carbon sources, as well as acetic acid, L-asparagine, L-glutamate, and D-alanine. Wild-type cells grew better on N-acetyl-D-glucosamine and D-mannose. Wild-type cells also grew better on L-histidine, the pyrimidines cytodine, cytosine, uridine, and thymidine, in addition to D-glucosamine, and N-acetyl-D-glucosamine as nitrogen sources (Panel B). The mutants grew better on L-asparagine and L-proline. In addition, several dipeptides provided differential growth conditions for wild-type cells and *flhD *mutants. No difference was observed between *flhD *and *fliA *mutants.

At 37°C, bacteria did not grow on the PM1 and PM3 plates. At 4°C, only three carbon sources provided better growth conditions for the wild-type cells (Panel C). These were D-mannose, D-mannitol, and sucrose. Several intermediates of the urea cycle provided better growth conditions for the mutants when used as single nitrogen sources (Panel D). These were ammonia, D-alanine, L-arginine, and L-proline. Uridine was the only one among the pyrimidines that still provided better growth conditions for wild-type cells. The dipeptides met-ala and gly-glu provided better growth conditions for the mutants. Again, there was no difference between the phenotypes of the *flhD *and the *fliA *mutants.

**Figure 2:**
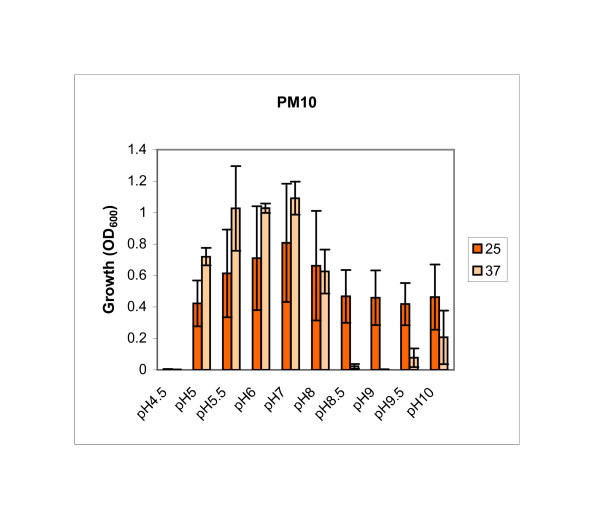
**Phenotype MicroArrays, pH**. Growth at different pH levels was determined with Phenotype MicroArrays (Biolog) for wild-type 8081c cells. Bacterial solutions in IF-10 were pipetted onto PM10 plates. Plates were incubated at 25°C (dark orange bars) or 37°C (light orange bars), growth was determined and is expressed as OD_630_. The experiment was performed three times, the mean of the population is presented. Error bars indicate 1 s.

In addition to the PM1 and PM3 plates, growth profiles of wild-type cells and *flhD *and *fliA *mutants were determined on PM10 plates. PM10 plates contain media of different pH, supplemented at low pH (4.5) with substrates of amino acid decarboxylases and at high pH (9.0) with substrates of amino acid deaminases. No difference was observed between wild-type cells and both mutants (data not shown). However, it is noteworthy that *Y. enterocolitica *can grow between a pH of 5 and 10 at 25°C (Fig. 2). At 37°C, all tested strains grew best between a pH of 5 and 8, but growth at pH 5 is better than at 25°C. This might be an adaptation to the human or animal host, where growth conditions are 37°C and the pH is low.

In summary, *flhD *and *fliA *mutants grew better on purines as carbon sources and wild-type cells grew better on pyrimidines as nitrogen sources and L-histidine. Carbon sources that are being degraded by glycolysis provided better growth conditions for wild-type cells. In contrast, carbon sources that are being degraded through the tricarboxylic (TCA) acid cycle provided better growth conditions for the mutants. Mutants also grew better than wild-type cells on intermediates of the urea cycle as nitrogen sources. The mutants did not have a phenotype on the pH plates that was different from the wild-type.

### Gene regulation correlates with the observed phenotypes

To correlate gene regulation with the expression of phenotypes, the levels of mRNA were determined for genes involved in the above metabolic pathways and possible transport systems (Table 1). These are the *hutC *and *hutU *operons for histidine utilization, the *carA *and *pyrB *operons for carbamoylphosphate synthesis and degradation, the *deoC *operon for purine metabolism, and the *acrE *operon for the pyruvate dehydrogenase complex. Remarkably, *Y. enterocolitica *has four operons for the transport of peptides [22]. Of particular interest are the two oligopeptide transport systems. The first one, *opp1*, contains a hybrid of *oppD *and *oppF *(the OppD/OppF hybrid protein contains two ABC_tran domains, but is lacking a C-terminal oligo_HPY domain [23]). The second one, *opp2*, contains a duplication of the *oppA *gene. Quantitative PCR (qPCR) was performed for selected genes (Table 2) with RNA isolated from cultures of wild-type 8081c cells and *flhD *and *fliA *mutants grown in LB at 25°C until an OD_600 _of 0.75.

**Table 1:** Annotation information for selected metabolic genes and operons

**Operon**	**Genes in operon**	**Function of the operon**
*fleC*	*fleC*	Flagellin
*hutC*	*hutC, hutI, hutG*	Histidine utilization
*hutU*	*hutU, hutH, hutP*	Histidine utilization
*carA*	*carA, carB*	Carbamoyl phosphate synthetase
*pyrB*	*pyrB, pyrI*	Aspartate carbamoyl transferase
*deoC*	*deoC, deoA, deoB, deoD*	Purine metabolism
*acre*	*acrE, lpdA, aceF*	Pyruvate dehydrogenase
*opp1*	*oppA1, oppB1, oppC1, oppD/F*	Oligopeptide transport
*opp2*	*oppA2, oppA2, oppB2, oppC2, oppD2, oppF2*	Oligopeptide transport
*dpp1*	*dppA1, dppB1, dppC1, dppD1, dpp1F*	Dipeptide transport
*dpp2*	*dppA2, dppB2, dppC2, dppD2, dppF2*	Dipeptide transport

Column 1 indicates the name of the operon that was analyzed.

Column 2 contains all genes in that operon.

Column 3 indicates the function of the operon in metabolism and/or transport. Annotation information was obtained from Sanger [22].

**Table 2:** Primers for qPCR

**Gene**	**Forward primer**	**Reverse primer**
*dnaE*	5'-AATCACACTCTGCCGCTTATG-3'	5'-CCATCCGCCAGCACTCAT-3'
*polA*	5'-GCTGGCTTGCGGATGTAGAT-3'	5'-AGCACGGCGGTCACTTCA-3'
*gapA*	5'-CCATCCGTGTTACCGCAGAG-3'	5'-TCTTAGCACCAGCAGCAATGT-3'
*fleC*	5'-GTAGTAGCGGTTTTCAGTGCTGT-3	5'-CCGGTGGTATTTCAGGTGAT-3'
*hutI*	5'-ACTCGCAGGTGTCACTTG-3'	5'-GAGCCAGTAAGCCAATTCAG-3'
*hutU*	5'-CGATATTCTTCCCAGCTCCA-3'	5'-GCAACAGATTTTGATGATGCG-3'
*carA*	5'-AAGGCAGTTGGACTCTGGAA-3'	5'-CCGTTGGATAAGAAAATGCC-3'
*carB*	5'-GTTAATTGGCGGCATTATGG-3'	5'-GACTTCAATCAGGTAAACTTC-3'
*pyrB*	5'-CCGATTGTCGTTTGAAACCT-3'	5'-ATTGACCACCGGCACATTGCC-3'
*deoB*	5'-GCTGACTGAAGGCGGTTACAAT-3'	5'-GGTGCTGGCGGTTCAACTG-3'
*acrE*	5'-AGATTCTGGCTAACGACTACG-3'	5'-ATCACCTGAGCGATGTATGG-3'
*oppA1*	5'-TCAAGAACAGCCAGAAGCCCTA-3'	5'-AAGCGAGCGTGGTTGGAATAC-3'
*oppB1*	5'-TGGCGGTGATAGTGGATT-3'	5'-TGATCTCGGCAATAACTTCC-3'
*oppC1*	5'-ACCAAGATCCGTATGTCATT-3'	5'-TACCGAGCCAGTTAGTGT-3'
*oppDF*	5'-TGAGATTATCCAGTGCCTTGA-3'	5'-TAGTCAGAACCGCCATAGC-3'
*oppB2*	5'-GCATTCCTACTGGCGGTGATT-3'	5'-AGGACTAACAGAGGAGCAACGA-3'
*dppB1*	5'-CCTTACTGACAGTGATTGC-3'	5'-ATAATGGCGGTGGTATCG-3'
*dppB2*	5'-ACCATTCCTCTCGCCGTTAT-3'	5'-GACCACAATCACTCGCATCC-3'

Column 1 presents the genes that were analyzed.

Columns 2 and 3 indicate the primers that were used for the qPCR reaction. Sequence information was obtained from Sanger [22].

**Figure 3:**
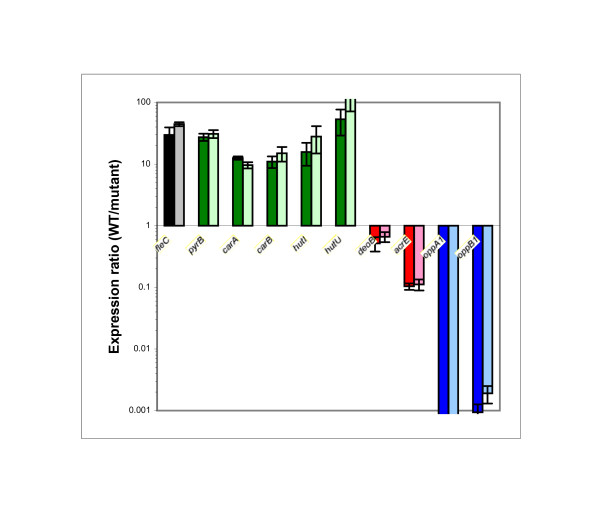
**Quantitative PCR**. Gene expression was determined for selected genes. Total RNA was isolated from wild-type 8081c cells and their isogenic *flhD *and *fliA *mutants. mRNA was reverse transcribed to cDNA and qPCR was performed. Data were normalized with *dnaE*, *polA*, and *gapA *(housekeeping genes). Expression ratios compare the expression levels between wild-type cells and *flhD *(darker bars) and *fliA *(lighter bars) mutants. Genes that are expressed higher in wild-type cells are printed in green, genes that are expressed higher in the mutants are printed in red, peptide transport genes are printed in blue. The *fleC *gene constitutes our internal control and is printed in black. The experiment was performed three times with each of three independently grown bacterial cultures and the mean of the population is presented. Error bars indicate 1 s.

The *fleC *gene (encoding flagellin) was used as a control for all qPCR experiments and exhibited an expression ratio of approximately 30 fold between the wild-type strain and the mutants (Fig. 3). This is consistent with previous studies [12,16]. The *pyrB*, *carA*, *carB*, *hutI*, and *hutU *genes were expressed at least ten times higher in the wild- type cells than in each of the mutants. In contrast, *deoB*, *acrE*, *oppA1 *and *oppB1 *were expressed higher in the mutants than in the wild-type cells. For the *oppA1 *and *oppB1 *genes, the expression ratio was more than 1000 fold. The *oppC1 *and *oppD/F *genes appeared unregulated by FlhD/FlhC and FliA, this was also seen in the remaining peptide transport systems (*oppB2*, *dppB1*, and *dppB2*) (data not shown).

In a previous study [12], FliA did not have an effect on the mRNA levels of the *pyrB*, *carA*, *carB*, *hutI*, and *hutU *genes. These data were obtained with RNA from cultures grown to an OD_600 _of 0.5. It appears that the growth phase of the culture has an impact on regulation. As one example, the class I operon *flhD *is expressed earlier in growth than the class II gene *fliA *[24-26]. The *pyrB*, *carA*, *carB*, *hutI*, and *hutU *genes seem affected by FlhD/FlhC in a FliA independent manner early in growth. Later in growth, FlhD/FlhC regulation of these genes did require FliA.

**Figure 4:**
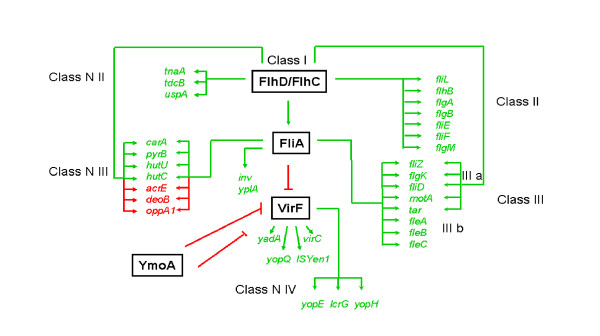
**Summary of the FlhD/FlhC and FliA regulated genes**. The cartoon demonstrates our current knowledge of *Y. enterocolitica *FlhD/FlhC and FliA dependent gene regulation [this study and [12,16,22,27,33-35]]. Regulators are printed in black and are surrounded by boxes. Regulated genes are printed in color. Genes that are positively regulated are printed in green (includes green arrow) and genes that are negatively regulated are printed in red (includes red arrow). The four classes of flagellar and non-flagellar genes are indicated.

Fig. 4 summarizes our current knowledge of gene regulation in *Y. enterocolitica *by FlhD/FlhC and FliA. As reported previously, the class I flagellar master regulator FlhD/FlhC regulates class II flagellar genes in a FliA independent manner and class III flagellar genes through FliA [12]. While class IIIb genes are regulated by FlhD/FlhC through FliA alone, class IIIa genes are also affected by FlhD/FlhC in a FliA independent manner. Regulation of metabolic genes is similar to the flagellar genes and a new classification for FlhD/FlhC regulated non-flagellar genes is proposed (Fig. 4). The *tnaA*, *tdcB*, and *uspA *operons (class N II) are regulated by FlhD/FlhC without any current evidence of FliA regulation [12,16]. The *carA*, *pyrB*, *hutU*, *hutC*, *acrE*, *deoC*, and *opp1 *operons (class N III) are regulated by FliA in late exponential growth (this study), possibly in addition to a FliA independent FlhD/FlhC dependent mechanism in mid-exponential growth [12,16]. In addition (class N IV), FliA represses a number of plasmid-encoded virulence genes [12], most likely through repression of their activator VirF [27]. Regulation of many plasmid-encoded virulence genes is an interplay between VirF [28-30] and the chromatin structure [31,32] that is believed to dislodge the histone-like protein YmoA [33]. The invasion gene *inv *and the phospholipase gene *yplA *are regulated by FliA [34,35].

**Figure 5:**
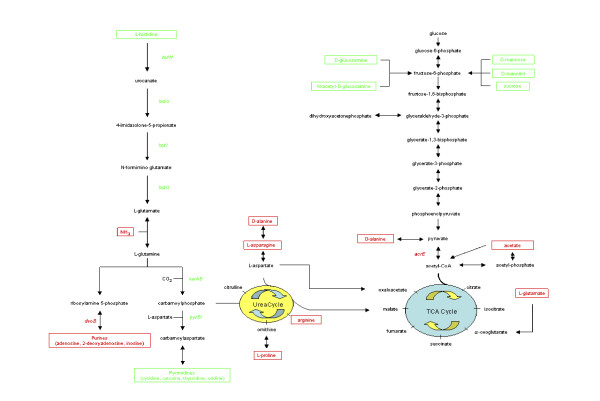
**Summary of the FlhD/FlhC and FliA regulated pathways**. The cartoon demonstrates our knowledge of *Y. enterocolitica *FlhD/FlhC and FliA affected pathways [this study and [16]]. Metabolites of major pathways are printed in black. Metabolites that feed into these pathways and provide better growth conditions for wild-type cells are printed in green. Metabolites that provide better growth conditions for the mutants are printed in red. Regulated genes are color coded accordingly. Data from the 25°C and 4°C experiments are combined and dipeptides are omitted.

Fig. 5 summarizes our current knowledge of FlhD/FlhC and FliA regulated pathways. L-histidine was one of the nitrogen sources, on which *flhD *and *fliA *mutants had exhibited a growth deficiency (Fig. 1). In agreement with this, several *hut *genes demonstrated higher expression in wild-type cells than in both mutants (Fig. 3). The *hut *genes serve histidine degradation in numerous bacteria, such as *Salmonella typhimurium *[36], *Klebsiella aerogenes *[37], and *Bacillus subtilis *[38]. Regulation of the *hut *genes by FlhD/FlhC and FliA (Fig. 3) likely explains the phenotype of reduced growth on L-histidine with the *flhD *and *fliA *mutants (Fig. 1).

The numerous differential phenotypes on dipeptides can most likely be explained with high regulaton of the Opp1 oligopeptide transport system. Other metabolic phenotypes are more difficult to understand. In addition to the flux of nitrogen through carbamoylphosphate that was postulated previously [16], FlhD/FlhC seems to affect the flux of carbon through pyruvate (Fig. 5). This hypothesis is supported by the observations that FlhD/FlhC and FliA support growth on carbon sources that are degraded through glycolysis, mainly fructose-6-phosphate [39], whereas carbon sources that are being degraded via the TCA cycle provide better growth conditions for the *flhD *and *fliA *mutants. In addition, the *acrE *gene, encoding one of the components of pyruvate dehydrogenase, was more highly expressed in the mutants than in the wild-type cells.

### Modification of the ELA for *Y. enterocolitica *strains

The group of genes that are included in Fig. 4 as regulated by FlhD/FlhC and have not been addressed yet are the virulence genes. This includes early virulence genes, such as *inv *and *yplA*, and late virulence genes, such as the *yop *genes. In an attempt to determine virulence of the *Y. enterocolitica flhD *and *fliA *mutants, we searched for a suitable animal model. The ELA is a virulence assay that was developed in an effort to determine the pathogenicity of *E. coli *strains that were isolated from healthy and diseased poultry. The assay correlated well with a number of phenotypic and genotypic traits that are associated with avian *E. coli *virulence [17-21]. In related studies, the ELA was compared to an intravenous chicken challenge method [18] and a subcutaneous challenge model [19]. A total of 20 strains yielded nearly identical results for the intraveneous challenge and the ELA [18]. So far, the ELA has been used solely to determine the virulence potential of *E. coli *isolates. In this study, we modified the well established assay for the use of *Y. enterocolitica *strains.

**Figure 6:**
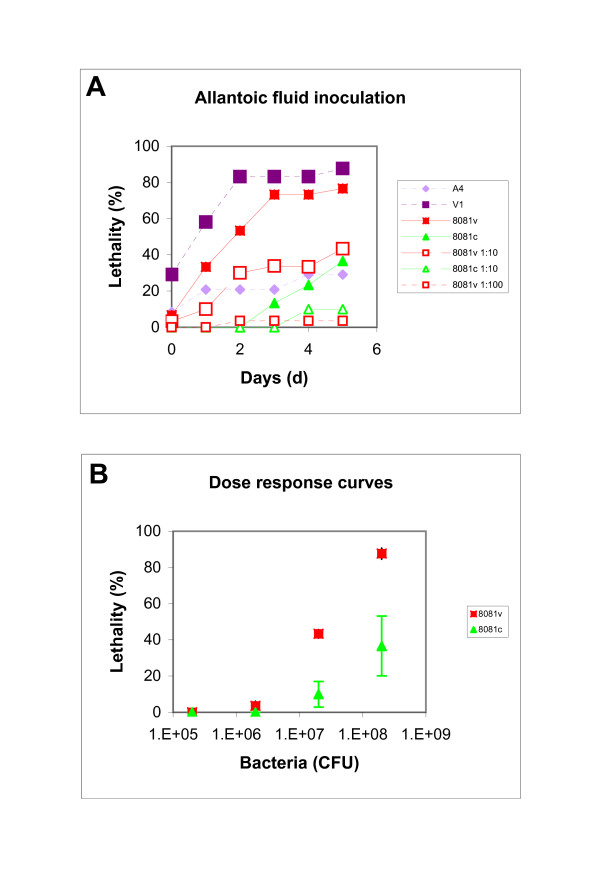
**Dose response curve for the ELA**. The ELA was performed with *Y. enterocolitica *8081v and 8081c strains and *E. coli *A4 and V1 strains as controls. Undiluted *Y. enterocolitica *cultures contained 2 × 10^9 ^CFU/ml and serial dilutions were performed in steps of 1:10. Bacterial cultures (100 μl) were administered to the chicken embryos into the allantoic fluid and lethality of the embryos was determined for five consecutive days (Panel A). The experiment was done twice, the mean of the average is presented. Standard deviations varied between 5 and 30%. Panel C is the dose response curve for the two *Y. enterocolitica *strains between inocula of 1 × 10^5 ^and 1 × 10^9 ^CFU after five days. Data are expressed as the mean over the two experiments and error bars indicate 1 s.

In the first experiment, we determined dose response curves for the *Y. enterocolitica *8081v and 8081c strains after allantoic fluid inoculation. Previously described *E. coli *strains [19] were used as positive and negative controls. Inoculation into the allantoic fluid yielded good lethality by the V1 (88%) and 8081v (77%) strains (Fig. 6A). Lethality caused by the A4 (29%) and 8081c (37%) strains was considerably lower. According to the dose response curve (Fig. 6B), an inoculum of 10^8 ^colony forming units (CFU) was chosen for *Y. enterocolitica *strains.

While the ELA has previously been shown to correlate with numerous virulence traits in *E. coli *[17-21], the common animal model that is being used for *Y. enterocolitica *is the mouse model [2,40]. The first target after orogastric infection of mice are the Peyer's patches of the small intestine [41,42]. Systemic infection involves the spleen, liver, and lungs, a progression that is believed to be similar to humans [43]. To compare the course of infection in chicken embryos with that in mice, we analyzed infected embryo organs and determined their bacterial load. A total of nine embryos (infected with 8081v) from two experiments were opened and the colonization of their internal organs was determined. Organs were colonized at 3.3 × 10^5 ^CFU/g for livers, 1.3 × 10^5 ^CFU/g for hearts, and 4.6 × 10^4 ^CFU/g for spleens. As a comparison, spleens of infected mice are colonized at approximately 10^4 ^CFU/g of organ after infection with an inoculum that is identical to ours at 10^8 ^CFU [40].

### Mutants in *flhD*, but not *fliA*, have reduced lethality in the chicken embryo lethality assay

**Figure 7:**
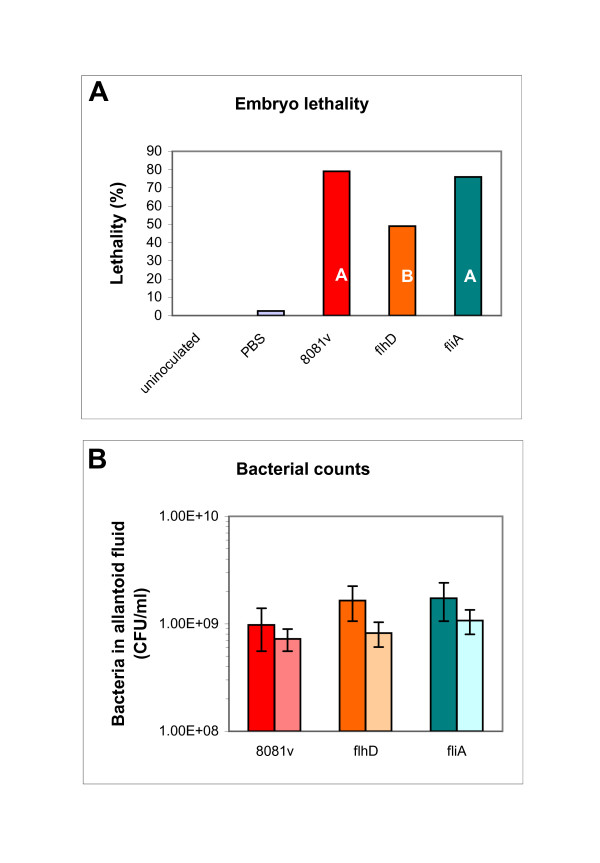
**Lethality of embryos inoculated with *Y. enterocolitica *strains (*flhD *and *fliA *mutants)**. The ELA was performed with *Y. enterocolitica *8081v, as well as with the *flhD *and *fliA *mutants in 8081v. Control groups of embryos were either uninoculated or inoculated with PBS. Embryos were inoculated with 10^8 ^CFU of the *Y. enterocolitica *strains. Lethality of the infected embryos was determined for five consecutive days. The experiment was performed three times. Panel A combines the number of dead embryos across the three experiments for each inoculum. Data are expressed as percentage of the total number of embryos for that inoculum. White capital letters in the *Y. enterocolitica *data bars represent the different groups from the Duncan's multiple group comparison. Panel B shows the bacterial counts in the allantoic fluid for dead (dark bars) and live embryos (light bars). Data are expressed as mean over the three replicate experiments and error bars indicate 1 s.

The pathogenic potential of the *Y. enterocolitica *8081v strain and its isogenic *flhD *and *fliA *mutants was determined with the ELA. Fig. 7A presents the lethality of the inoculated embryos, calculated over three experiments. This includes the two control groups of embryos (uninoculated and PBS) as a comparison. Lethality of the embryos inoculated with the 8081v strain was highest at 79%, lethality caused by the *fliA *mutant was similar (76%). The *flhD *mutant exhibited reduced lethality of 49%. When comparing 8081v to the *flhD *mutant, the odds ratio resulting from the logistic regression analysis for dead embryos was 0.189. This indicates that the likelihood of death was approximately 5 times higher for embryos that were inoculated with the 8081v strain than with the *flhD *mutant strain. Comparison of the *fliA *mutant to 8081v yielded an odds ratio of 0.772, indicating that there was no statistically significant difference between the 8081v strain and the *fliA *mutant. This result was confirmed with the Duncan's multiple group comparison, which found two significantly different groups. The A group contains the 8081v strain and the *fliA *mutant, the *flhD *mutant forms its own group B (Fig. 7A).

Fig. 7B represents the bacterial counts from the allantoic fluid of the embryos that were inoculated with the *Y. enterocolitica *strains. The average of bacterial counts for each test group of embryos was between 9 × 10^8 ^and 1.7 × 10^9 ^bacteria/ml of allantoic fluid for dead embryos and 6 × 10^8 ^to 1 × 10^9 ^bacteria/ml of allantoic fluid for living embryos. While living embryos contained generally somewhat lower numbers of bacteria, differences in growth between the four strains were minor and did not parallel differences in lethality (Fig. 7A). In addition, growth of the strains in liquid LB was very similar for all three strains (8081v, 1.28 gen/h; 8081v *fliA*, 1.25 gen/h; 8081v *flhD*, 1.2 gen/h). Obviously, differences in lethality (Fig. 7A) can not be explained by differences in bacterial growth (Fig. 7B). This is consistent with a previous study using the ELA for *E. coli *strains [20].

The difference in the lethality caused by 8081v and 8081c parallels observations from a mouse model [43]. In this model, the 8081v strain killed mice within 5 days of oral infection and the 8081c strain was unable to kill mice or establish systemic infection [44]. The association of flagella with infection has been demonstrated for several gram-negative bacteria [45-47]. Specifically, mutants of avian *E. coli*, defective in flagella synthesis, were significantly less able to persist in one day old chicken [48]. Also, flagella were required for persistent infection of chickens by *E. coli *O157:H7 [49]. This could explain our results with the *flhD *mutant, but not with the *fliA *mutant, as neither mutant synthesizes flagella. Results from the *fliA *mutant parallel a previously published experiment with mice [2].

Our data indicate that the effect of the *flhD *mutant on virulence is not due to genes that are regulated by FlhD/FlhC in a FliA dependent manner (such as flagellin genes and plasmid-encoded virulence genes), but instead indicates the genes that are regulated by FlhD/FlhC in a FliA independent manner (class II, class N II; Fig. 4)). One of such genes is the *flhB *gene that encodes one protein of the flagellar specific type III secretion system that is responsible for the transport of certain flagellar proteins [for reviews, please, see [7,50]] and a number of virulence factors [51].

**Figure 8:**
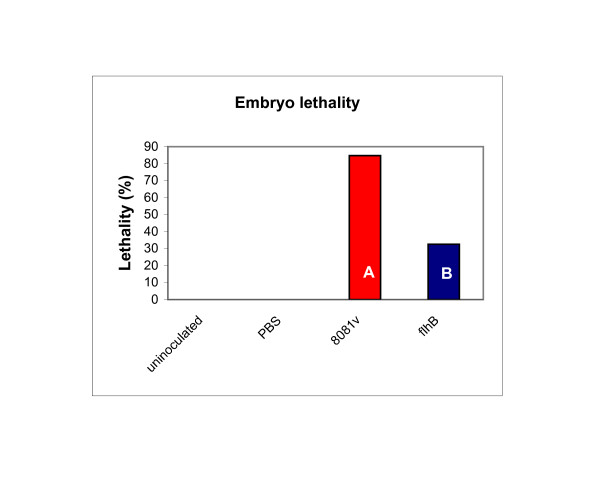
**Lethality of embryos inoculated with *Y. enterocolitica *strains (*flhB *mutant)**. The ELA was performed with *Y. enterocolitica *8081v and its isogenic *flhB *mutant. Control groups of embryos were either uninoculated or inoculated with PBS. Embryos were inoculated with 10^8 ^CFU of the *Y. enterocolitica *strains. Lethality of the infected embryos was determined for five consecutive days. The experiment was performed three times. The Figure combines the number of dead embryos across the three experiments for each inoculum. Data are expressed as percentage of the total number of embryos for that inoculum. White capital letters in the *Y. enterocolitica *data bars represent the different groups from the Duncan's multiple group comparison.

We repeated the ELA, comparing the lethality caused by a *flhB *mutant to that caused by 8081v (Fig. 8). In three experiments, the odds ratio of lethality for the *flhB *mutant relative to 8081v was 0.069, indicating that the odds ratio of dying from 8081v was 14 to 15 times higher than for the *flhB *mutant. The Duncan's multiple group comparison yielded two statistically different groups. These results are similar to the *flhD *mutant and consistent with a hypothesis that the lethality phenotype of the *flhD *mutant was due to genes that are regulated by FlhD/FlhC in a FliA independent manner, such as the ones that encode the flagellar type III secretion system.

Interestingly, possession of the *flhD *operon might not be the only requirement for virulence of *Y. enterocolitica*. The genes also may have to be properly regulated. First, cultures of 8081v that had grown only into mid-exponential phase (OD_600 _of 0.5 to 0.9) had a much reduced lethality (30%) when compared to cultures that had grown into stationary phase (OD_600 _higher than 1.0). It is possible that these cultures had not reached their maximum motility yet. Second, a plasmid that permitted constitutive expression of *flhD *did not complement the *flhD *mutant (odds ratio of 1.266 between 8081v *flhD *and 8081v *flhD *pGY17). FlhD/FlhC is a positive regulator of early virulence genes and a repressor of late virulence genes. Therefore, constitutive expression of the *flhD *operon would not allow for the expression of the late virulence genes. Apparently, full virulence of *Y. enterocolitica *requires *flhD *to be regulated in a way that allows for expression of the early virulence genes at the time of infection, and expression of the late virulence genes later during the infection process.

## Conclusion

Our phenotypic analysis yielded pleiotropic phenotypes of *Y. enterocolitica flhD *and *fliA *mutants. These correlate with previously and newly described gene regulations. Phenotypes include growth differences (between the wild-type cells and the mutants) on several nutrients used as sole carbon or nitrogen sources. Among the phenotypes that can easily be explained by the respective gene regulation is the deficiency of the mutants to grow on L-histidine. This phenotype is likely caused by the simultaneous reduction in the expression level of the *hut *genes. A dramatic increase in the expression level of the *opp1 *operon in the mutants probably explains differences between wild-type cells and mutants in the ability to grow on several dipeptides. Pyruvate was postulated as a check-point for the flux of carbon that is affected by FlhD/FlhC.

The second investigated phenotype was virulence, as determined with a chicken embryo model. In this model, *flhD *mutants, but not *fliA *mutants, caused a reduced lethality among the embryos when compared to their isogenic wild-type strain. The phenotype of the *fliA *mutant is consistent with a previous mouse model [2]. The phenotype of the *flhD *mutant correlates with previous gene regulation [12]. The observation that a mutant in *flhB *also exhibited a reduced embryo lethality indicates that the lethality phenotype of the *flhD *mutant might be due to the lack of the flagellar type III secretion system.

## Methods

### Strains and growth conditions

The two *E. coli *isolates, V1 and A4, are poultry isolates that were extensively characterized and used to establish and refine the ELA [17-19]. These two isolates were used as positive (virulent; V1) and negative (avirulent; A4) controls. *Y. enterocolitica *8081 is one of the American serotypes of *Y. enterocolitica*, which are human pathogens causing systemic infections more frequently than gastroenteritis [52,53]. The serotype O:8 is virulent for mice, the infection affects the mesenteric lymph nodes, the liver and the spleen [43]. The 8081v strain contains the pYV virulence plasmid, whereas the 8081c strain has been cured of this plasmid [54,55]. The remaining *Y. enterocolitica *strains are derivatives of 8081v, containing mutations in the flagellar regulator operons, *flhD, fliA*, and *flhB *[14]. The *flhD *operon encodes the master regulator complex FlhD/FlhC [1,3]. The *fliA *gene encodes the flagellar specific sigma factor FliA [2,10]. The *flhB *operon encodes components of the flagellar type III secretion system. The mutants were constructed through insertional inactivation of the respective gene in a reverse genetics approach, using the streptomycin Ω cassette. All *Y. enterocolitica *strains have been kindly provided by Dr. Scott A. Minnich (University of Idaho, Moscow ID). Plasmid pGY17 (*flhD *and *flhC*, [1]) was kindly provided by Dr. Virginia L. Miller (Washington University School of Medicine, St. Louis MO) and conjugated into 8081v. Selective antibiotics were 10 μg/ml tetracycline to select for the presence of the plasmid and 40 μg/ml of nalidixic acid to counter-select the *E. coli *donor strain. The resulting strain SMH999 exhibits wild-type levels of motility and possesses the pYV virulence plasmid.

Cultures of all bacterial isolates and strains were maintained as freezer stocks (-70°C) in 1 ml of Luria Bertani broth (LB: 1 % tryptone broth, 0.5 % NaCl, and 0.5 % yeast extract), containing 15% glycerol or DMSO. *Y. enterocolitica *8081v strains were inoculated into liquid LB straight from the freezer stock and incubated over night at 25°C in order to avoid plasmid loss. All other strains were plated onto agar plates prior to the experiment. For Phenotype MicroArrays, bacteria were plated onto R2A plates, as recommended by the manufacturer. For all other experiments, bacteria were inoculated into liquid LB and incubated overnight at the designated temperature.

### Phenotype MicroArrays

Phenotype MicroArrays™ (PMs) were obtained from Biolog (Hayward, CA). These are 96 well plates with a different nutrient dried to the bottom of each well [56]. The media are designed to test a multiplicity of cellular phenotypes. An indicator dye turns purple upon transferral of electrons that are produced during respiration. In this study, we used PM1, PM3 and PM10 plates. PM1 plates contain carbon sources, PM3 plates contain nitrogen sources and PM10 plates were designed for pH screening. The plates were inoculated with 100 μl of a cell solution that was standardized with the 85% turbidity standard. Cell suspensions for PM1 and PM3 plates were prepared in IF-0 GN Base inoculation fluid, cell suspensions for PM10 plates in IF-10 GN Base. Sodium pyruvate (20 mM) was added as a carbon source to cell suspensions that were used for PM3 plates. Dye A was used as an indicator dye for all plates as recommended by the manufacturer.

Growth was monitored with a Synergy AT plate reader from Biotek Instruments (Winooski, VT) at 630 nm. Growth at 25°C was analyzed after 24 h, a second reading was taken at 48 h only for PM3 plates. Data for cytosine, uridine, met-ala, and ala-leu are presented from the 48 h reading (Fig. 1B), all other data are from the 24 h reading. Growth at 4°C was analyzed after 240 h. The OD_630 _of the wild-type culture was divided by the OD_630 _of the mutant after subtraction of the background in field A1 which contained only the medium without a nutrient. Growth ratios above 1 indicate nutrients that provide better conditions for the wild-type cells. Growth ratios below 1 indicate nutrients that provide better conditions for the mutant. Each experiment was done at least three times and means and standard deviations were determined. A threshold level of a significant difference in growth was defined as growth above 2 or below 0.5, in agreement with previous work [12,16,57]. Only nutrients that allowed one of the strains to grow to an OD_630 _of at least 0.3 were considered for analysis.

### Quantitative PCR

For each experiment, RNA was isolated from three independently grown bacterial cultures. RNA was isolated with the hot phenol/SDS method [58], final cleaning of the RNA was performed with an RNeasy mini column (Qiagen, Valencia, CA). RNA samples were treated with DNaseI (Qiagen) twice. RNA was converted to cDNA using the protocol described by Patrick O. Brown [64].

The qPCR was performed with the iQ SYBR Green Supermix from BioRad (Hercules, CA). The reaction mixture contained 30 – 60 ng cDNA, 50 mM KCl, 20 mM Tris-HCl pH 8.4, 0.2 mM of each dNTP, 0.625 U iTaq DNA polymerase, 3 mM MgCl_2_, SYBR Green I, 10 nM fluorescein, and 100 nM of each primer (Table 2). Primer pairs were previously described [12,16] or newly designed with Beacon Designer 4.0 (Premier Biosoft International, Palo Alto CA). The enzyme was activated for 3 min at 95°C. Reactions were performed in triplicate at 94°C for 30 sec, 55°C for 30 sec and 72°C for 1 min (50 ×) and monitored in an iCycler iQ qPCR detection system (BioRad). Melting curves were obtained for each reaction and generally contained single peaks. The cDNA dilution (0.01 ng to 10 ng) curves indicated increases in the PCR product concentrations between 1.85 and 2.1 fold per cycle.

Different techniques to normalize expression ratios obtained from qPCR experiments have been discussed [59,60]. In this study, qPCR data were normalized with three housekeeping genes, *dnaE*, *polA*, and *gapA*. These three genes are generally perceived as constitutively expressed with respect to numerous conditions [61,62]. Their expression levels were not affected by FlhD/FlhC or FliA previously [12,16]. Differential threshold cycle crossings (ΔTc) for each gene were corrected by the average difference in Tc for the three housekeeping genes to yield (ΔTc_norm_). Expression ratios were calculated as 2^-ΔTcnorm^. All expression ratios are expressed as mean and standard deviation, obtained from the triplicate measurements of cDNA samples that were obtained from the three independently grown bacterial cultures.

Statistical analysis of the data after the second normalization was performed with the *z*-test which identifies significant differences between a sample mean (expression ratios) and a set number of 1 (fold). Based upon the low *p*-values that we obtained for all of our genes, we believe that all these genes are regulated by FlhD/FlhC and FliA.

### Embryo lethality assay

Overnight cultures grown at 25°C in LB were washed once with phosphate buffered saline (PBS: NaCl, 8 g/l; KCl, 0.2 g/l; Na_2_HPO_4_, 1.44 g/l; K_2_HPO_4_, 0.24 g/l; pH 7.2) and diluted in 100 μl of PBS. The number of bacteria used for inoculation was 10^6 ^CFU for the *E. coli *strains and 10^8 ^CFU for the *Y. enterocolitica *strains. The dose response curves for the *Y. enterocolitica *strains were done between 10^5 ^and 2 × 10^8 ^CFU. The number of bacteria inoculated into the embryos was verified using the viable count method [18]. The presence of the pYV virulence plasmid in the inocula derived from the 8081v series of strains was confirmed with congo red plates [63]. Bacteria containing the pYV plasmid yielded small pin-point colonies of dark red color on these plates, whereas bacteria that had lost the plasmid yielded larger, less colorful colonies.

Specific pathogen free chicken embryos were purchased from Sunrise Farms, Inc. (Catskill, NY). Upon arrival, the embryos were incubated in a Sportsman incubator, model #1502 (G.Q.F. MFG. CO, Savannah, GA) at 37°C. Humidity was kept constant at 86%. On day twelve after setting, the allantoic fluids of the embryonated eggs were inoculated with the above indicated number of bacteria. Each strain was tested in the ELA in two (dose response curve) or three to four (all other experiments) independent experiments with test groups of embryos of at least 5 (dose response curve) or 10 (all other experiments). Two control groups of embryos (uninoculated and PBS-inoculated embryos) were included in each experiment. After inoculation, incubation was continued for a maximum of seven days. Humidity and temperature of the incubator were checked daily. The embryos were candled each day and the number of deaths was recorded. Data from day 1 were omitted since we observed random death across all test groups of embryos, probably due to trauma from the inoculation. Lethality was expressed as percent of the total number of embryos in each test group. The allantoic fluid of dead embryos was cultured. At the end of the experiment, all remaining eggs were cultured for bacteria. Recovered bacterial counts in the allantoic fluid were typically in the 10^8 ^to 10^9 ^CFU/ml range for all bacteria used.

### Statistical analysis of the ELA data

Because percentage data is not a continuous variable nor is it normally distributed, the percent lethality resulting from each isolate was subjected to a logistic regression analysis. Specifically, odds were calculated for each strain as (p/(1-p)) with p representing the percentage of lethality as determined in the ELA. This was done for each experiment individually. Odds ratios across multiple experiments were determined, using the 8081v strain as baseline reference strain for the logistic regression analysis. Odds ratios presented in the Results section are the result of this regression.

Lethality of several test groups of embryos that were inoculated with the *Y. enterocolitica *strains was also compared with a Duncan's Multiple Group Comparison. This test determines whether differences in lethality between the test groups are statistically significant. All statistical analyses were performed on SAS v 9.1 (SAS Institute Inc., Cary, NC, USA.).

### Preparation of chicken embryo organs

Organs were prepared and tested for bacteria as described [40]. Briefly, we removed livers, hearts, and spleens from 4 to 5 surviving embryos from two of the ELA experiments. All of these embryos had been infected with the 8081v wild-type strain and incubated for five days post infection. Organs were washed twice with 1 ml of PBS and weighed. Livers were minced and stomached in 10 ml of PBS, hearts and spleens in 5 ml. Stomaching was performed in a Stomacher 400 Circulator from Seward (West Sussex, Great Britain) for 2 min. A total of 10 μl of suspension was removed from each bag and serially diluted in steps of 1:10. Bacteria were plated onto LB plates. Bacterial counts were determined after incubation at 25°C overnight and calculated as CFU/g of organ.

## Abbreviations

CFU: Colony forming units; DMSO: Dimethylsulfoxide; ELA: Embryo lethality array; LB: Luria Bertani broth; PBS: Phosphate buffered saline; PCR: Polymerase chain reaction; PM: Phenotype MicroArray; qPCR: Quantitative PCR; TCA: Tricarboxylic acid.

## Authors' contributions

MKT is a graduate student in the laboratory of BMP. She performed the PM10 arrays, the qPCR, the *flhD *and *fliA *ELA, and the dose response curves. NJC and JGY are undergraduate students in the laboratory of BMP. They performed the PM3 and PM1 arrays, respectively. SMH is a research associate in the laboratory of BMP. She did the complementation experiment. PSG and BMP are assistant professors in the Department of Veterinary and Microbiological Sciences. PSG has more than 10 years of experience with the ELA and taught everybody the technique. She also performed some of the allantoic fluid inoculations and helped interpreting the statistical analysis of the ELA data. BMP designed and supervised the project and wrote the manuscript. She also performed the *flhB *ELA, the organ preparations, and the remaining allantoic fluid inoculations.

All authors have read and approved the manuscript.
